# Microbes Tickling Your Tummy: the Importance of the Gut-Brain Axis in Parkinson’s Disease

**DOI:** 10.1007/s40473-017-0129-2

**Published:** 2017-11-08

**Authors:** Paula Perez-Pardo, Mitch Hartog, Johan Garssen, Aletta D. Kraneveld

**Affiliations:** 1 Division of Pharmacology, Utrecht Institute for Pharmaceutical Sciences, Faculty of Science, Utrecht University, Universiteitsweg 99, 3584 CG Utrecht, The Netherlands; 2 Nutricia Research, Utrecht, The Netherlands; 3 Institute for Risk Assessment Sciences, Faculty of Veterinary Medicine, Utrecht University, Utrecht, The Netherlands

**Keywords:** Parkinson’s disease, Gastrointestinal dysfunction, Gut microbiota, Inflammation, Alpha-synuclein

## Abstract

**Purpose of Review:**

Patients suffering from Parkinson’s disease (PD) are known to experience gastrointestinal dysfunction that might precede the onset of motor symptoms by several years. Evidence suggests an important role of the gut-brain axis in PD pathogenesis. These interactions might be essentially influenced by the gut microbiota. Here, we review recent findings supporting that changes in the gut microbiota composition might be a trigger for inflammation contributing to neurodegeneration in PD.

**Recent Findings:**

Recent research revealed that PD patients exhibit a pro-inflammatory microbiota profile in their intestinal tract that might increase gut permeability, allowing leakage of bacterial products and inflammatory mediators from the intestines. Evidence in literature indicates that alpha-synuclein deposition might start in the enteric nervous system by pro-inflammatory immune activity and then propagates to the CNS. Alternatively, the peripheral inflammatory response could impact the brain through systemic mechanisms.

**Summary:**

A better understanding of the gut-brain interactions and the role of the intestinal microbiota in the regulation of immune responses might bring new insights in PD pathological progression and might lead to novel diagnostics and therapeutic approaches.

## Introduction

Parkinson’s disease (PD) is the second most common age-related neurodegenerative disease, with increasing age being the greatest risk factor for its development. PD is hallmarked by the loss of dopaminergic neurons in the substantia nigra (SN), resulting in the characteristic motor impairments. These symptoms are commonly treated with dopamine-replacement medication, but these therapies do not prevent the dopaminergic neurodegeneration. Another relevant hallmark in PD is the presence of alpha-synuclein-containing inclusion bodies (Lewy pathology) in the surviving neurons in different areas of the nervous system [1–3].

Besides the well-known motor deficits, PD patients very often suffer from non-motor symptoms including hyposmia, anxiety, depression, impaired executive function, and most commonly gastrointestinal (GI) dysfunction [4, 5, 6•]. Some of these symptoms are very often present in pre-clinical stages, and their occurrence in healthy individuals is associated with an increased risk of developing PD [4, 7, 8].

Recent evidence suggest an important role of the GI tract and the associated enteric nervous system (ENS) in the development of PD [9–12]. The so-called gut-brain axis is a bidirectional communication system between the central nervous system (CNS) and the GI tract. The immune system might be an important interplayer by regulating immune homeostasis in both the gut and the brain [13]. It has been also lately recognized that the gut-brain interactions might be essentially influenced by the gut microbiota [14–16].

The cause of neuronal death in PD remains a matter of debate; however, abundant evidence suggests that inflammatory mechanism might play an important role. For instance, in the brain, microglial activation is associated with dopaminergic neuronal loss suggesting that neuroinflammation may contribute to the degenerative process [17]. Moreover, it has been reported that alpha-synuclein has an important role in the initiation and maintenance of inflammation in PD [18]. The early involvement of the GI tract in PD supports the hypothesis that the GI tract could be a source of inflammation contributing to neurodegeneration.

## The Role of the GI Tract in PD

### GI Dysfunction in PD

Recent evidence suggests that GI dysfunctions are consistently associated with PD. The occurrence and prevalence of different GI dysfunctions vary among patients and has been extensively reviewed [6•]. Among GI problems, constipation is the most prominent (reported in 20 to 80% of PD patients) and it is partially due to a prolonged intestinal transit time affecting both the small intestine and the colon [6•, 19]. Constipation might precede motor symptoms by over a decade [6•, 8, 19, 20] making it one of the earliest indicators of a pathological process that will ultimately lead to PD. Recently, constipation was included in the research criteria for prodromal PD diagnostics as one of the risk factors for future development of PD [21].

### Alpha-Synuclein Pathological Progression

Alpha-synuclein is a protein expressed as a normal component of the CNS and the ENS and is thought to be involved in the regulation of neurotransmission and synaptic homeostasis [22, 23]. Under certain circumstances, alpha-synuclein aggregates, leading to one of the pathological hallmarks in PD, Lewy bodies in cell somata, and Lewy neurites in axons and dendrites [24, 25], were found in the brain of individuals with PD. It is not entirely clear if Lewy bodies are toxic for the neurons or if they are formed in an attempt to sequester alpha-synuclein aggregates to avoid damage of cellular components and cytotoxicity. What is clear is that the overexpression of alpha-synuclein has been proven to be sufficient to induce its aggregation and the degeneration of dopaminergic neurons [26].

Several studies indicated that alpha-synuclein is also detected in higher levels in the intestines of PD patients compared to healthy controls [27–30, 31••, 32]. These alpha-synuclein accumulations are associated with damage in the enteric neurons and possibly underlie GI dysfunction [27, 32].

The spatial distribution of alpha-synuclein aggregates of PD patients is generally compatible with a staging scheme proposed in 2003 postulating that inclusions initially appear in the olfactory bulb (OB) and dorsal motor nucleus of the vagus (DMV) in the lower brainstem [33••]. The pathology then seems to spread in an ascending fashion with the subsequent involvement of monoaminergic pontine nuclei and the dopaminergic neurons of the SN. In accordance with the hypothesis, it has been proven that alpha-synuclein exhibits prion-like properties, including the ability to misfold and form aggregates, which display cell-to-cell transmission [34, 35].

Braak and colleagues postulated in their “dual-hit hypothesis” that the initial formation of alpha-synuclein aggregates occurs outside the brain, in the terminals of neurons residing in the olfactory bulb and the ENS as a consequence of exterior insults such as toxins and/or microorganisms [33••, 36–38]. The vagus nerve might provide a path for the spread of alpha-synuclein pathology from the ENS to the brain through the brainstem, midbrain, basal forebrain, and finally the cortical areas [36, 39], whereas the initiation of the pathological process in the OB might ultimately affect the brain via the olfactory tract [11, 37, 40]. Recent studies [31••, 41••] suggest that gut-initiated pathological processes in PD do not necessarily require an environmental pathogen and/or toxin since they can be triggered by the commensal bacteria in the gut.

Factors such as microorganisms, including nasal/gut microbiota, and toxins such as pesticides or pollutants might be the initial inflammatory trigger in the nasal and intestinal mucosa, inducing damage and the subsequent mucosal inflammation and oxidative stress, thereby initiating alpha-synuclein accumulation.

### Pre-clinical and Clinical Evidence

Alpha-synuclein has the capability of spontaneous misfolding and shows prion-like properties, including cell-to-cell propagation [34, 42, 43]. Both retro- and anterograde axonal transports of alpha-synuclein fibrils have been shown to occur via the vagal nerve [44, 45], and pathological aggregates were detected in the DMV when alpha-synuclein fibrils were injected in the duodenum of rats [46]. Moreover, a single intra-peritoneal injection of alpha-synuclein fibrils in A53T transgenic mice led to marked neurological PD-like symptoms and alpha-synuclein pathology in the spinal cord and the brain [47]. In another transgenic mouse model for PD, alpha-synuclein was shown to be transmitted to engrafted neuronal precursor cells, where it created inclusions [48, 49]. Similarly, autopsies of PD patients that received fetal mesencephalic transplants displayed alpha-synuclein accumulation in the grafted neurons [50, 51].

If, indeed, the vagal nerve constitutes a major path for alpha-synuclein pathology spreading, vagotomy might then be protective against PD development. In 2015, the first epidemiological study tested whether vagotomy modifies the risk of PD. Cohorts of patients, who had undergone vagotomy between 1977 and 1995, were compared to matched controls. Full truncal vagotomy was associated with a decreased risk of developing PD compared to super-selective vagotomy (affecting only acid producing portion of gastric body) or no vagotomy [52•].

This finding has now been reproduced in a Swedish cohort where the full truncal vagotomy group exhibited a decreased risk of developing PD after more than 5 years of follow-up when compared with the background population and also when compared to the selective vagotomy group (affecting vagal branches to the stomach) and the super-selective vagotomy group [53].

Observations in animal models also support that the resection of parts of the autonomic nervous system delays the progression of alpha-synuclein pathology. A pre-clinical study demonstrated that PD-like pathology could be mimicked by the oral administration of the pesticide rotenone in mice [54]. The local effect of pesticides on the ENS induced PD-like progression and reproduced the neuroanatomical and neurochemical features of PD staging from the ENS to the brain [54]. The same group showed that the progression of alpha-synuclein pathology towards the brain could be halted by the resection of sympathetic and parasympathetic nerves before the oral administration of rotenone [9]. Both the olfactory and intestinal mucosae are exposed to substances from the environment via inhalation or ingestion, and therefore, it seems probable that these environmental factors such as diets, pesticides, pollutants, and mucosal microorganisms might have an important role in triggering PD pathology, probably supported by genetic predisposition.

### Gut Leakiness

Several studies have demonstrated that PD patients have increased intestinal permeability, also known as “leaky gut,” in comparison to healthy controls [31••, 55–58]. Interestingly, intestinal permeability has been shown to correlate with the levels of enteric alpha-synuclein [31••]. Alpha-synuclein aggregates might be formed as a consequence of oxidative injury [26]. Deficits in the gut barrier function and the translocation of bacteria and inflammatory bacterial products (e.g., lipopolysaccharide, LPS) might trigger an inflammatory response and the associated oxidative stress in the gut and thereby initiating alpha-synuclein accumulation in the ENS [31••, 59–61] that might ultimately reach the brain in a “prion-like” manner. Furthermore, gut-derived LPS can promote the disruption of the blood-brain barrier [62, 63] and thus contribute to neuroinflammation and injury in the SN.

### Changes in Gut Microbiota Composition

The increased intestinal permeability has been detected in recently diagnosed PD patients [31••], but no studies have yet assess intestinal permeability in healthy individuals that will later develop PD. Therefore, we do not have evidence supporting that increased intestinal permeability is a pre-motor stage but the symptom is present at least in the early stages of the disease.

Results from different studies have shown that the gut microbiota is altered in PD patients.

Small intestinal bacterial overgrowth (SIBO) is a malabsorption syndrome associated with increased bacterial density and/or the presence of colonic-type species in the small intestine [64]. SIBO is highly prevalent in PD patients [32, 65], even when recently diagnosed [66]. The abnormalities in GI motility developed in PD patients might be responsible for the increased occurrence of SIBO. However, once SIBO is developed, it might increase intestinal permeability and contribute to bacterial translocation and therefore perpetuating the inflammatory response [67].

Recently, gut microbiota content in different cohorts of PD patients was characterized in several studies. The first study describing differences in the composition of fecal bacteria populations reported an increased abundance of *Enterobacteriaceae* in PD that positively correlated with the severity of postural instability and gait difficulty [68••]. They further detected lower abundance of *Prevotella*, prominent producers of health-promoting neuroactive short chain fatty acids (SCFA) as well as important promoters of the biosynthesis of thiamine and folate [69], which is in line with decreased levels of these vitamins in PD patients [70, 71]. The authors further indicate that a decrease in *Prevotella* might be related with a reduction in mucin synthesis which is associated with increased intestinal permeability [31••, 65] intensifying the translocation of bacterial antigens. In addition, a decreased abundance of *Prevotella* and an increased abundance of *Lactobacillaceae* have been associated with lower concentrations of ghrelin. Ghrelin is a gut hormone that may be involved in the maintenance and protection of normal nigrostriatal dopamine function [72], and impaired ghrelin secretion has been reported in PD patients [73].

A second study revealed differences in both mucosal and fecal microbial communities of PD patients in comparison to healthy subjects. More differences were found in fecal than in mucosal (tissue adherent) bacteria [41••]. The study showed a lower abundance of SCFA butyrate-producing bacteria associated with anti-inflammatory properties from the genera *Blautia*, *Coprococcus*, and *Roseburia* in PD fecal samples, thereby concluding that a reduction in SCFA might contribute to gut leakiness. In addition, metagenomics analysis indicated that genes involved in LPS biosynthesis and type III bacterial secretion systems were higher in stool samples of PD patients compared to controls. Type III secretion systems are involved in pathogenic interactions with host cells and might exacerbate bacterial product-induced inflammation [74, 75]. PD pathogenesis may be caused or exacerbated by changes in GI microbiota composition that could induce peripheral inflammatory responses and ultimately promote alpha-synuclein pathology in the intestine and the brain or by rostral to caudal cell-to-cell transfer of alpha-synuclein pathology caused by increased oxidative injury (due to an increase in pro-inflammatory bacteria).

SCFA concentrations have also been demonstrated to be reduced in PD patients compared to age-matched controls that exceed the reductions normally associated with aging [76]. Significant reduction of acetate, propionate, and butyrate were observed in PD fecal samples. The reduction in SCFA might contribute to the reduction of peristaltic regulation by the ENS and contribute to GI dysmotility in PD.

Furthermore, SCFA butyrate has anti-inflammatory properties thought to be owing to an epigenetic mechanism or to the activation of SCFA receptors leading to anti-/microbial and anti-inflammatory effects and to a decreased intestinal barrier dysfunction [77–80].

More recently, the analysis of the gut microbiota in a German cohort revealed differences in relative abundance for four bacterial families in PD patients in comparison to controls, including an increase of *Lactobacillaceae* which is in accordance with the previous studies [81].

There are several variables that could affect the microbiota and cover up the disease signature. Since most PD patients are using medication, it is important to differentiate the disease effects from that of medication. In a case-only analysis, besides dysbiosis of the gut microbiota in PD patients, significant differences were found in gut microbiota composition as a function of treatment with COMT inhibitors and anticholinergics [82]. The authors indicate that the effects of COMT inhibitors and anticholinergics are independent of the PD effect because their impact on the overall microbiome was detected within patients (hence, PD was controlled for). Moreover, most of the PD-associated taxa were robustly associated with disease in patients who were not on either of these drugs. It was not possible to determine the effects of carbidopa/levodopa since 90% of the patients are taking this medication.

The gut microbiota might be involved in the metabolism of the drugs used by PD patients and might affect its absorption. The drugs might in turn affect microbiota composition.

So far, it is not possible to determine if changes in the gut microbiota are an occurrence contributing to the disease initiation or if they emerge as a consequence of PD-related pathology. However, it might still play a role in neurological dysfunction and neurodegeneration by perpetuating inflammatory cascades and oxidative injury in the brain through LPS-mediated mechanism. The microbiota, via mechanisms including metabolite production, can impact immune and inflammatory pathways leading to the peripheral and central immune activation and inflammation [83, 84]. Colonic biopsies retrieved from PD patients revealed an increased expression in the levels of pro-inflammatory cytokines, such as TNF-alpha, IFN-gamma, IL-6, and IL-1 beta as well as an increased activation of enteric glial cells [85]. Moreover, IBD and IBS, two chronic inflammatory conditions, have been reported to increase the risk of PD development [86, 87].

The importance of the intestinal microbiome in PD was recently demonstrated in a study showing that under germ-free conditions, or when bacteria are depleted with antibiotics, mice overexpressing human alpha-synuclein displayed reduced microglia activation, alpha-synuclein inclusions, and motor deficits compared to animals with a complex microbiota. These results suggest that gut microbiota is required for PD phenotype development. Moreover, reconstitution of the fecal microbiota in germ-free alpha-synuclein transgenic PD mice using stool from PD patients exacerbates motor symptoms and pathology in this model [88••].

## Proposed Mechanism for PD Pathology

New evidence suggests that gut-derived inflammation may play a mechanistic role in PD pathogenesis [89–99]. As previously described, GI dysfunctions are very common in PD and might begin decades before the onset of motor symptoms [4, 7, 8]. The intestinal tract is a large surface area in direct contact with microorganisms [100] that have the capability to provoke inflammatory responses. Several groups reported that PD patients exhibit a pro-inflammatory microbiota profile [41••, 68••, 76] that might cause and increase in gut permeability, allowing leakage of bacterial products and inflammatory mediators from the intestines.

**Fig. 1:**
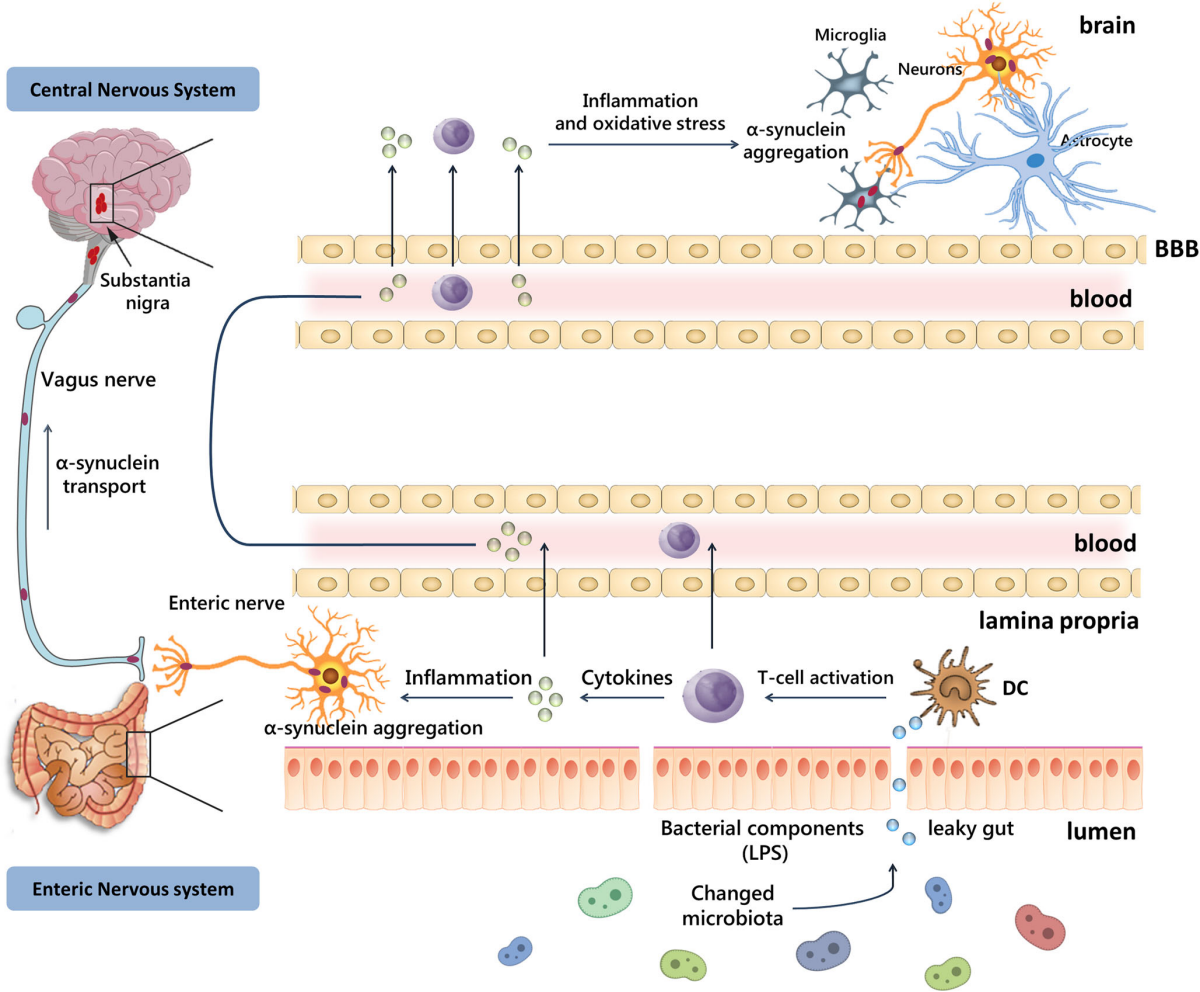
Possible pathways involved in PD pathogenesis. Changes in the gut microbiota composition might increase intestinal permeability, allowing leakage of bacterial products and inflammatory mediators from the intestines. Alpha-synuclein deposition might start in the ENS by pro-inflammatory immune activity and then propagates to the CNS. Alternatively, the peripheral inflammatory response could impact the brain through systemic mechanisms. In the brain, alpha-synuclein can activate microglia that might already been activated due to the ongoing gastrointestinal and systemic immune responses.

Current evidences indicate that alpha-synuclein deposition in PD might start in the ENS by pro-inflammatory immune activity [101, 102] and propagates to the CNS by transsynaptic cell-to-cell transmission. Overexpressed and aggregated alpha-synuclein would in turn stimulate pro-inflammatory responses from creating a vicious cycle [102, 103]. Alternatively, gut-derived bacterial products or the peripheral inflammatory response (e.g., cytokine production) could impact the brain through systemic mechanisms including disruption of the blood-brain barrier, which is observed in PD patients [104]. In the brain, alpha-synuclein activates microglia [102] that might already been activated due to the ongoing gastrointestinal and systemic immune responses. Within the brain, dopaminergic neurons are particularly sensitive to inflammation [102] and might begin to degenerate. When there is enough depletion of striatal dopamine as the results of the loss of these dopaminergic neurons, motor dysfunction will start manifesting (Fig. 1).

## Conclusion

PD and possibly other neurodegenerative diseases are associated with dysbiosis in the GI tract, which can result in an unbalance of the host immune system. As a consequence, mucosal and systemic inflammation might ultimately reach the brain where they cause neurodegeneration. Lately, it has been hypothesized that PD pathology starts in the GI tract decades before progressing to the CNS. Therefore, a better understanding of the gut-brain interactions and the role of the intestinal microbiota in the regulation of immune responses might bring new insights in PD pathological progression as well as lead to novel diagnostics and therapeutic approaches. For instance, the modulation of the gut microbiota using pre-, pro-, and antibiotics or fecal transplantations might, in the future, be a viable therapeutic strategy for PD.
